# Organizational factors associated with work-related sleep problems in a nationally representative sample of Korean workers

**DOI:** 10.1007/s00420-012-0759-3

**Published:** 2012-03-17

**Authors:** Jae Bum Park, Akinori Nakata, Naomi G. Swanson, Heekyoung Chun

**Affiliations:** 1 Department of Occupational and Environmental Medicine, School of Medicine, Ajou University, Suwon, South Korea; 2 Division of Applied Research and Technology, National Institute for Occupational Safety and Health, Centers for Disease Control and Prevention, 4676 Columbia Parkway, Cincinnati, OH 45226 USA; 3 Education and Information Division, National Institute for Occupational Safety and Health, Centers for Disease Control and Prevention, 4676 Columbia Parkway, Cincinnati, OH 45226 USA

**Keywords:** Sleep problems, Work organization, Korean Working Conditions Survey, Psychosocial job characteristics, Korea

## Abstract

**Purpose:**

The purpose of this study was to assess the association of organizational factors with work-related sleep problems (WRSP) among Korean workers.

**Methods:**

The data were derived from the First Korean Working Conditions Survey conducted in 2006 with a representative sample of the Korean working population (*n* = 10,039).

**Results:**

The overall prevalence of WRSP was 5.1  % (95  % confidence interval (CI) 4.7–5.5). Those who experienced sexual harassment at work (adjusted odds ratio (aOR) 3.47: 95 % CI 1.77–6.81), discrimination due to sex (aOR 2.44: 95 % CI 1.36–4.36) or age (aOR 2.22: 95 % CI 1.52–3.23), violence at work (aOR 1.98: 95 % CI 1.06–3.68), threat of violence (aOR 1.96: 95 % CI 1.05–3.66), poor work-life balance (aOR 1.78: 95 % CI 1.44–2.20), low job satisfaction (aOR 1.69: 95 % CI 1.37–2.09), high cognitive (OR 1.64: 95 % CI 1.32–2.03) and emotional (aOR 1.53: 95 % CI 1.22–1.91) demands, job insecurity (aOR 1.32: 95 % CI 1.07–1.63), and high work intensity (aOR 1.55: 95 % CI: 95 % CI 1.25–1.92) had an increased risk of WRSP compared to their respective counterparts (*p* < 0.01). Low social support was not significantly associated with WRSP (aOR 0.88: 95 % CI 0.67–1.15).

**Conclusion:**

The results revealed that poor psychosocial working conditions may be related to a high prevalence of WRSP among representative Korean workers.

## Introduction

Sleep problems have been one of the most commonly reported health complaints associated with a variety of physical and mental health outcomes (Ohayon 2002). According to a global estimate of sleep problems based on 10 different countries (*n* = 35,327), 31.6 % of individuals suffer from insomnia and 24.0 % report that they do not sleep well (Soldatos et al. 2005). In Korea, the prevalence of sleep problems ranges between 5.0 and 32.9 % (Cho et al. 2009; Kim et al. 2011; Nomura et al. 2010; Ohayon and Hong 2002), depending on the characteristics of the population sampled and the definition/case assessment. Sleep problems including insomnia, daytime sleepiness, and sleep apnea have been linked to a variety of occupational health issues; those employees with sleep problems reported an elevated risk of depression and anxiety disorders (Motohashi and Takano 1995; Nakata 2011b), alcohol abuse (Weissman et al. 1997), suicide (Goldstein et al. 2008) and chronic pain (Kuppermann et al. 1995) and are considered to be at high risk for hypertension (Murata et al. 2007; Yang et al. 2006) and coronary heart disease (Mallon et al. 2002).

Sleep problems have a profound negative impact not only for individuals but also for the workplace and society as a whole. Consequences of sleep problems include reduced productivity (Nena et al. 2010; Rosekind et al. 2010), increased injuries at work (Kling et al. 2010; Lombardi et al. 2010; Nakata 2011a; Salminen et al. 2010; Vahtera et al. 2006), absenteeism (Akerstedt et al. 2010; Nakata et al. 2004b; Philip et al. 2001), and medical care expenditures (Leger and Bayon 2010; Metlaine et al. 2005). To date, a number of studies have examined the relationship between work organization factors and sleep problems; these studies have identified overtime work (Dahlgren et al. 2006), job dissatisfaction (Nakata et al. 2004a, 2007; Scott and Judge 2006), overcommitment (Kudielka et al. 2004; Ota et al. 2005), effort-reward imbalance (Fahlen et al. 2006; Ota et al. 2005, 2009), low job control (Runeson et al. 2011), high job demands (Cahill and Landsbergis 1996; Kalimo et al. 2000; Knudsen et al. 2007; Nakata et al. 2007; Pelfrene et al. 2002; Runeson et al. 2011), role conflict (Knudsen et al. 2007), poor interpersonal relationships (Nakata et al. 2004a, 2007) job insecurity (Ferrie et al. 1998; Kim et al. 2011), workaholism (Kubota et al. 2010), and poor social support from colleagues/supervisors (Nakata et al. 2001, 2004a; Ota et al. 2009; Pelfrene et al. 2002; Runeson et al. 2011; Sinokki et al. 2010), as risk factors for sleep problems, although earlier studies have emphasized the negative impact of non-standard work schedules, that is, shift/night work, on sleep (Akerstedt et al. 2002; Estryn-Behar et al. 1990; Niedhammer et al. 1994). In addition, emerging workplace issues, that is, workplace bullying (Lallukka et al. 2011; Niedhammer et al. 2009; Takaki et al. 2010), violence at work (Eriksen et al. 2008), and occupational injustice (Elovainio et al. 2009; Kim et al. 2011), are found to be strongly related to sleep problems.

Although previous studies have suggested that work organization and the nature of work are associated with sleep problems, a few have drawn that conclusion based on representative samples of workers. The data from the National Employment Survey 2002–2003, a nationally representative random sample of 1,715 US full-time employees, indicated that work overload and repetitive work were associated with difficulty initiating and maintaining sleep while work overload and role conflict were related to non-restorative sleep (Knudsen et al. 2007). In addition, most previous studies used self-administered questionnaires rather than face-to-face interviews, which may introduce reporting/recall bias (Bowling 2005). Furthermore, little information is available about Korean workers; a study by Kim et al. (2011), which used a self-administered questionnaire, reported that high job demands, insufficient job control, inadequate social support, job insecurity, organizational injustice, lack of reward, discomfort with the occupational climate, and overall job stress were related to a 13–45 % increased risk of insomnia (Kim et al. 2011).

Based on the above facts, continued effort is needed to explore the relationship between work organization factors and sleep problems. Therefore, this study was undertaken to investigate the relationship between work organization factors and sleep problems in a large nationally representative sample of Korean workers using data collected via face-to-face interviews.

## Methods

### Subjects and procedure

Data were derived from the First Korean Working Conditions Survey (KWCS), conducted in 2006 by the Korea Occupational Safety and Health Agency (KOSHA) (Park and Lee 2009). The survey population was a representative sample of the actively working population aged 15–65 years (in Korea, the legal work age is 15 years). ‘Economically active’ refers to subjects who were either employees or self-employed at the time of interview. Therefore, those who were retired, unemployed, housewives, or students were not included in the survey. The basic study design was a multistage random sampling of the enumeration districts used in the 2005 population and housing census (Park and Lee 2009). Data collection was performed by Gallup Korea during June 26 to September 26, 2006. A total of 46,498 households were visited, and 10,043 interviews were performed. A total of 36,515 households had dropped out of the interview. The number of households where a member of the household could not be interviewed after visiting 3 times was 14,680, while the number of households where a member of household was encountered but was not qualified to be a respondent was 2,671. The number of households without an employed person aged between 15 and 64 (non-qualified household) was 12,192, and the number of households that refused to take part was 6,972. We excluded workers who were under 18 (*n* = 4), which resulted in a final sample size of 10,039 respondents.

The survey weighting was carried out on the basis of the actively working population, which means that its distribution by age, sex, region, locality, size, economic activity, and occupation is identical to that of the active population distribution. Sociodemographic characteristics of the sample and total working population in Korea are shown in Table 1, suggesting that the distributions of the KWSC and the Korean total working population are comparable. The questionnaire contains questions about hours of work, physical risk factors, work organization, and the impact of work on health. The methodology and survey questionnaire of the first KWCS were based on that of the Fourth European Working Conditions Survey (EWCS) in 2005 (Parent-Thirion et al. 2006).

**Table 1:** Demographic characteristics of the participants in Korean Working Condition Survey, 2006

	Sample ( %)^a^	Population ( %)
Age group		
15–24	5.4	7.4
25–34	23.3	23.7
35–44	32.0	27.7
45–54	25.0	23.5
55–	14.3	17.6
Sex		
Men	57.9	58.0
Women	42.1	42.0
Education		
Below middle school	19.7	24.3
High school	41.4	42.4
College/university and beyond	38.9	33.3
Industry sectors		
Agriculture, forestry and fishing	7.4	8.3
Mining and manufacturing	21.2	17.9
Construction	6.5	7.9
Wholesale and retail trade, hotels, and restaurants	19.8	24.8
Electricity, transport, telecom. and finance	11.4	10.0
Education	8.4	7.2
Other services	25.4	24.0
Total number	“10,043”	“23,447,000”

^a^Figures of sample population are weighted

### Variables

#### Sleep problems

Sleep problems in this study were assessed by the single item ‘Do you currently suffer from work-related sleep problems (WRSP)?’ which is identical to the question used in the EWCS. The response was either ‘yes’ or ‘no.’

#### Work organization factors

Descriptions of work organization factors, response options, and response criteria are shown in Table 2. In all, 12 work organization variables were included in the questionnaire. The subjects were asked to answer ‘yes’ or ‘no’ about their experiences of discrimination regarding age and sex, sexual harassment, threat of violence, and violence at work during the past 12 months. Job insecurity, cognitive work demands, and emotional work demands were measured with a five-point scale. Job satisfaction and work-life balance were measured with a four-point scale. Social support at work and work intensity were measured by the sum of two items, both with five-point scales. The Cronbach’s α for social support at work and for work intensity was 0.87 and 0.83, respectively.

According to the report provided by KOSHA (Park and Lee 2006), the test–retest reliability for the 1-month interval for the items ‘working at very high speed,’ ‘working too tight deadlines,’ and ‘intellectually demanding work’ had 60.1, 61.7, and 68.5 % consistency rates, respectively.

**Table 2:** Questions regarding work organizational factors

Variables	Questions and response options (in parentheses)	Response criteria
Sexual harassment	Over the past 12 months, have you been subjected to sexual harassment at work? (yes/no)	Yes
Sexual discrimination	Over the past 12 months, have you been subjected to sexual discrimination at work? (yes/no)	Yes
Age discrimination	Over the past 12 months, have you been subjected to age discrimination at work? (yes/no)	Yes
Threat of violence	Over the past 12 months, have you been subjected to threats of physical violence at work? (yes/no)	Yes
Violence at work	Over the past 12 months, have you been subjected to physical violence at work? (yes/no)	Yes
Work-life balance	In general, do your working hours fit in with your family or social commitments outside work? (very well, well, not very well, not at all well)	Not very well, not at all well
Job satisfaction	On the whole, how satisfied are you with your working conditions in your main paid job? (very satisfied, satisfied, not very satisfied, not at all satisfied)	Not very satisfied, not at all satisfied
Cognitive demands	You find your job intellectually demanding. (almost always, often, sometimes, rarely, almost never)	Almost always, often, sometimes
Emotional demands	You find your job emotionally demanding. (almost always, often, sometimes, rarely, almost never)	Almost always, often
Work intensity	(a) Do you work at very high speed? and (b) Do you work too tight deadlines? [never (0 % of time), almost never (10 % of time), about 25 % of time, about 50 % of time, around 75 % of time, almost all the time (90 % of time), always (100 % of time)]	Median split: high (36–200), low (0–35)
Job insecurity	I might lose my job in the next 6 months. (strongly agree, agree, neither agree nor disagree, disagree, strongly disagree)	Strongly agree, agree
Social support	(a) You can get assistance from colleagues if you ask for it and (b) You can get assistance from supervisors if you ask for it (almost always, often, sometimes, rarely, almost never)	Rarely, almost never

#### Other potential confounding variables

Potentially confounding variables were sex, age group (15–24, 25–34, 35–44, 45–54, and 55+), educational level, income per month (<1 [≈€ 820.34], 1–3, or >3 [≈€ 1,640.69] million Korean won), smoking status (never, former, current), and alcohol consumption (number of alcoholic drinks consumed/day, with one drink estimated as about 9 g of pure ethanol). Symptoms related to work included depression, anxiety, muscular pain, backache, headache, injuries, stomachache, eyesight problems, skin problems, hearing problems, allergies, and heart disease. Other variables included job type classified into 10 categories according to the Korean Standard Classification of Occupation (Statistics Korea 2007), type of employment (employed, self-employed, or employer), working hours per week (<35, 35–44, or ≥45), employment contract (full-time or part-time), and work schedule (daytime or shift/night).

#### Statistical analyses

A series of univariate and multiple logistic regression analyses were conducted individually to examine the associations of organizational factors with sleep problems. All the work organization variables were dichotomized into two groups as suggested in Table 2. First, we tested the relationship between potential confounders and sleep problems with univariate analyses and then with forward stepwise multiple logistic regression analysis (*p* ≤ 0.05 for inclusion and *p* ≥ 0.10 for exclusion). Second, to examine the relationship between organizational factors and sleep problems, multiple logistic regression analyses were used and were incrementally adjusted for sex, age group, highest education level, and income (Model A); Model A variables + smoking status, alcohol consumption, and presence of illnesses (Model B); Model B variables + employment status, job type, employment contract, working hours, and work schedule (Model C). The significance level for all statistical analyses was *p* < 0.05 (two-tailed test).

## Results

The characteristics of the study participants are shown in Table 3. There were 5,809 male and 4,230 female workers. The prevalence of sleep problems was 5.1 % (95 % CI: 4.7–5.5 %). Participants ranged in age from 18 to 65 (mean 42) years. More than one-third held a college degree or higher and 62 % earned a monthly income of 1–3 million Korean won. Overall, 32 % were current smokers, 13.9 % were former smokers, and more than 70 % were current alcohol drinkers. About a quarter of the workers reported one or more physical symptoms/disorders, almost 30 % were self-employed or an employer, and 7.2 % of participants worked a shift/night schedule. The four dominant job types were professional/technical (19.1 %), clerical (14.0 %), service (12.4 %), and sales (11.4 %). More than half of the participants worked 45 h or more per week.

**Table 3:** Characteristics of study population (*n* = 10,039)

Characteristics	*n* ( %)
Work-related sleep problems (yes)	510 (5.1)
Sex	
Male	5,809 (57.9)
Female	4,230 (42.1)
Age (years), mean (SD)	42 (10.9)
Age group, years	
18–24	544 (5.4)
25–34	2,338 (23.3)
35–44	3,213 (32.0)
45–54	2,511 (25.0)
55–65	1,433 (14.3)
Highest education	
Below middle school	1,979 (19.7)
High school	4,157 (41.4)
College/university and beyond	3,903 (38.9)
Smoking status	
Never	5,425 (54.0)
Former	1,396 (13.9)
Current	3,218 (32.1)
Alcohol consumption (g ethanol/week)	
Non-drinker	2,837 (28.3)
0.01–49.9	3,508 (34.9)
50.0–99.9	1,247 (12.4)
100.0–299.9	1,866 (18.6)
>300.0	581 (5.8)
Presence of illness	
No	7,561 (75.3)
Yes	2,478 (24.7)
Employment status	
Employed	7,092 (70.6)
Self-employed or employer	2,947 (29.4)
Income (million Korean won/month)	
<1 (€ 820.34)^a^	2,574 (25.6)
1–1.99	4,061 (40.4)
≥2 (€ 1,640.69)	3,404 (33.9)
Job type	
Senior manager	244 (2.4)
Professional/technical	1,913 (19.1)
Clerical	1,409 (14.0)
Service	1,249 (12.4)
Sales	1,141 (11.4)
Agriculture/fisheries	779 (7.8)
Skilled	1,053 (10.5)
Machine operator	1,107 (11.0)
Unskilled	1,101 (11.0)
Armed forces	43 (0.4)
Employment contract	
Full-time work	9,651 (96.1)
Part time	388 (3.9)
Working hours per week	
<35	1,012 (10.1)
35–44	3,137 (31.2)
≥45	5,885 (58.6)
Missing	5 (0.1)
Work schedule	
Non-shift (daytime)	9,306 (92.7)
Shift/night	728 (7.2)
Missing	5 (0.1)

^a^At an exchange rate of approximately 1,219 Korean won per €1 (as of Aug 1, 2006)

The covariates associated with sleep problems are shown in Table 4. The univariate logistic regression analyses revealed that male gender, older age (≥55), current smoking, higher alcohol consumption, presence of illness, job type, long working hours (≥45 h/week), and shift/night work were significant factors associated with sleep problems. In the stepwise multivariate logistic regression analyses, male gender, age group, presence of illness, job type, and shift/night work schedule remained significant.

**Table 4:** Associated factors underlying risk of work-related sleep problems in a representative sample of Korean workers (*n* = 10,039)

Characteristics	Univariate OR		Multivariate OR^a^	
	(95 % CI)	*p* value	(95 % CI)	*p* value
Sex		<0.001		<0.001
Female	1.00		1.00	
Male	1.51 (1.25–1.82)		1.53 (1.21–1.93)	
Age group, years				
18–24	1.00	<0.001	1.00	0.028
25–34	1.47 (0.88–2.46)		1.35 (0.76–2.40)	
35–44	1.63 (0.99–2.69)		1.29 (0.73–2.28)	
45–54	1.39 (0.83–2.32)		0.88 (0.49–1.57)	
55–65	2.39 (1.43–4.00)		1.26 (0.69–2.31)	
Highest education		0.031		
Below middle school	1.36 (1.07–1.72)			
High school	1.06 (0.86–1.30)			
College/university and beyond	1.00			
Income (million Korean won/month)		0.177		
<1 (€ 820.34)	1.00			
1–1.99	1.11 (0.89–1.38)			
≥2 (€ 1,640.69)	1.33 (0.99–1.78)			
Smoking status		<0.001		
Never	1.00			
Former	1.91 (1.50–2.43)			
Current	1.44 (1.18–1.76)			
Alcohol consumption (g ethanol/week)		0.039		
Non-drinker	1.00			
0.01–49.9	1.29 (1.01–1.63)			
50.0–99.9	1.36 (1.00–1.84)			
100.0–299.9	1.30 (0.99–1.71)			
>300.0	1.72 (1.19 2.49)			
Presence of illness		<0.001		<0.001
No	1.00		1.00	
Yes	81.4 (53.3–124.4)		82.6 (53.8–126.7)	
Type of employment		<0.001		
Employed	1.00			
Self-employed or employer	1.64 (1.37–1.97)			
Job type		<0.001		<0.001
Senior manager	1.84 (0.90–3.67)		1.84 (0.82–4.09)	
Professional/technical	1.82 (1.22–2.73)		1.36 (0.87–2.12)	
Clerical	1.00		1.00	
Service	2.46 (1.62–3.72)		1.67 (1.04–2.68)	
Sales	2.10 (1.34–3.19)		1.38 (0.85–2.24)	
Agriculture/fisheries	4.68 (3.11–7.05)		1.45 (0.89–2.38)	
Skilled	2.14 (1.38–3.31)		0.83 (0.51–1.34)	
Machine operator	3.53 (2.36–5.28)		1.01 (0.64–1.61)	
Unskilled	1.11 (0.67–1.83)		0.64 (0.37–1.10)	
Armed forces	1.03 (0.15–7.16)		0.35 (0.05–2.73)	
Employment contract		0.372		
Full time	1.00			
Part time	1.26 (0.76–2.01)			
Working hours (hours/week)		0.019		
<35	1.00			
35–44	0.81 (0.56–1.16)			
≥45	1.47 (1.07–2.04)			
Work schedule		<0.001		<0.001
Non-shift	1.00		1.00	
Shift/night	2.75 (2.15–3.52)		2.54 (1.86–3.47)	

*OR* odds ratio, *CI* confidence interval

^a^Forward stepwise multiple logistic regression analysis (*p* ≤ 0.05 for inclusion and *p* ≥ 0.10 for exclusion)

The relationships between psychosocial work characteristics and sleep problems are shown in Table 5. Univariate logistic regression analyses showed that all 12 organizational variables were significantly associated with a 25–525 % increased prevalence of sleep problems. After controlling for covariates, social support at work did not remain significant, but the rest of the 11 variables remained significant.

**Table 5:** Organizational factors underlying work-related sleep problems in the representative sample of Korean workers (*n* = 10,039)

Organizational factors:	*n* ( %)		Crude	*p* value	Model A^a^	*p* value	Model B^b^	*p* value	Model C^c^	*p* value
Sexual harassment	9,976 (99.4)	No	1.00	<0.001	1.00	<0.001	1.00	0.001	1.00	<0.001
	63 (0.6)	Yes	6.25 (3.49–11.2)		6.99 (3.87–12.6)		3.11 (1.61–6.00)		3.47 (1.77–6.81)	
Sexual discrimination	9,894 (98.6)	No	1.00	<0.001	1.00	<0.001	1.00	0.005	1.00	0.003
	145 (1.4)	Yes	3.02 (1.86–4.91)		3.79 (2.31–6.21)		2.27 (1.29–3.99)		2.44 (1.36–4.36)	
Age discrimination	9,696 (96.6)	No	1.00	<0.001	1.00	<0.001	1.00	<0.001	1.00	<0.001
	343 (3.4)	Yes	3.21 (2.33–4.42)		3.38 (2.44–4.69)		1.94 (1.35–2.78)		2.22 (1.52–3.23)	
Violence at work	9,964 (99.3)	No	1.00	<0.001	1.00	<0.001	1.00	0.006	1.00	0.032
	75 (0.7)	Yes	6.09 (3.55–10.4)		6.01 (3.49–9.14)		2.30 (1.17–4.16)		1.98 (1.06–3.68)	
Threat of violence	9,959 (99.2)	No	1.00	<0.001	1.00	<0.001	1.00	0.007	1.00	0.035
	80 (0.8)	Yes	5.27 (3.07–9.05)		5.30 (3.09–9.14)		2.26 (1.25–4.09)		1.96 (1.05–3.66)	
Work-life balance	7,268 (72.4)	Good	1.00	<0.001	1.00	<0.001	1.00	<0.001	1.00	<0.001
	2,771 (27.6)	Poor	3.07 (2.57–3.68)		3.02 (2.51–3.63)		1.96 (1.61–2.40)		1.78 (1.44–2.20)	
Job satisfaction	6,712 (66.9)	High	1.00	<0.001	1.00	<0.001	1.00	<0.001	1.00	<0.001
	3,327 (33.1)	Low	3.52 (2.90–4.27)		3.44 (2.84–4.17)		1.76 (1.43–2.16)		1.69 (1.37–2.09)	
Cognitive demands	5,365 (53.4)	Low	1.00	<0.001	1.00	<0.001	1.00	<0.001	1.00	<0.001
	4,674 (46.6)	High	1.79 (1.49–2.15)		1.94 (1.61–2.34)		1.61 (1.31–1.98)		1.64 (1.32–2.03)	
Emotional demands	5,578 (55.6)	Low	1.00	<0.001	1.00	<0.001	1.00	<0.001	1.00	<0.001
	4,461 (44.4)	High	1.71 (1.42–2.04)		1.90 (1.58–2.21)		1.54 (1.26–1.89)		1.53 (1.22–1.91)	
Work intensity	5,270 (52.5)	Low	1.00	<0.001	1.00	<0.001	1.00	0.001	1.00	<0.001
	4,769 (47.5)	High	2.27 (1.88–2.74)		2.32 (1.92–2.81)		1.44 (1.17–1.78)		1.55 (1.25–1.92)	
Job insecurity	6,540 (65.1)	Low	1.00	0.017	1.00	0.015	1.00	0.032	1.00	0.009
	3,499 (34.9)	High	1.25 (1.04–1.50)		1.26 (1.05–1.51)		1.25 (1.02–1.53)		1.32 (1.07–1.63)	
Social support at work	5,845 (65.9)	High	1.00	0.014	1.00	0.128	1.00	0.718	1.00	0.348
	4,194 (34.1)	Low	1.26 (1.05–1.51)		1.16 (0.96–1.41)		1.04 (0.84–1.29)		0.88 (0.67–1.15)	

*OR* odds ratio, *CI* confidence interval

^a^Adjusted for age group, sex, educational level, and income

^b^Adjusted for age group, sex, educational level, income, smoking, drinking, and presence of illness

^c^Adjusted for age group, sex, educational level, income, smoking, drinking, presence of illness, type of employment, type of occupation, employment contract, working time, and work schedule

## Discussion

The purpose of this study was to investigate the relationship between work organization factors and WRSP in a large representative sample of Korean workers. There were three key findings from this study. First, organizational factors related to violence, discrimination, work-life imbalance, job dissatisfaction, high work demands and intensity, and job insecurity were associated with an increased prevalence of WRSP even after adjusting for an array of potential confounders. Second, male gender, age group, presence of illness, and shift/night work were background risk factors associated with high WRSP prevalence. Third, the overall prevalence of WRSP was 5.1 % in this population. Although the results must be interpreted with caution because of the cross-sectional nature of the study design, the analyses of this large population-based representative survey suggest that work organization factors are important risk factors for WRSP among Korean workers.

Those who experienced sexual harassment at work had a 3.5 times higher risk of WRSP compared to those who had not experienced sexual harassment at work. Although we could not locate studies specifically focused on a relationship between sexual harassment and workers’ sleep problems, several studies have reported the relationship between sexual harassment and workers’ physical and mental health. A study on female flight attendants showed that for those who experienced sexual harassment, the risk of poor self-rated health was 2.8 times higher than for those who had not had such an experience (Ballard et al. 2006). There are also reports that sexual harassment heightens the risk of depression, somatic symptoms, posttraumatic stress disorder (PTSD), and other medical conditions (Street et al. 2008), which could relate to sleep problems. Sexual harassment also raises the risk of the victims’ harmful alcohol use (Gradus et al. 2008). Given such evidence, workers who experienced sexual harassment may have an increased risk for suffering sleep problems.

This study found that the participants who perceived sex- and age-related discrimination had more than twice the risk of WRSP than those workers who did not. Discrimination is a crucial social issue not only in multiethnic nations such as the United States but also in non-multiethnic nations as well. In the United States, the occurrence of perceived discrimination over one’s lifetime is 33.5 %, but the prevalence differs greatly by racial/ethnic group; for non-Hispanic whites, it is 30.9 %, for non-Hispanic blacks, 48.9 %, and for other racial/ethnic groups, 50.2 % (Kessler et al. 1999). The results of the 1977–1989 US Longitudinal Survey of Mature Women (*n* = 1,778) indicated that perceived workplace discrimination ranged between 11.11 and 15.14 % in black women, while it ranged between 12.10 and 16.03 % in white women. Workplace discrimination was found to be one of the strongest predictors for emotional distress and functional limitation (Pavalko et al. 2003). In the current study, the occurrence of age and sex discrimination at the workplace was 3.4 and 1.4 %, respectively, which was lower than those of studies conducted in the United States (Kessler et al. 1999; Pavalko et al. 2003), but the impact on sleep seems substantial. Taking these findings into consideration, workplace discrimination may have a long-lasting negative influence on sleep behavior, possibly resulting in poor mental health of affected workers.

Additionally, in this study, those who experienced violence at their work sites were twice as likely to suffer from sleep problems as those who did not. A study of Nurses’ aides revealed that those who had been exposed to threats or violence at work had a 19 % increased risk of poor sleep compared to those without such exposures (Eriksen et al. 2008). With fear acting as a mediator, the experience of violence is known to adversely affect workers’ health both mentally and physically (Rogers and Kelloway 1997). Even when an individual is not a direct victim of violence, being a witness to a threatening act has been reported to exert negative effects (anxiety, illness symptoms, and negative occupational outcomes) (Hall and Spector 1991). The result of this study corresponds with the notion and that workers who are exposed to threats of violence had an equivalent risk of sleep problems as those who actually had undergone violence at work.

Work-life imbalance has become an emerging issue in Korea because of an increase in working hours (Park et al. 2010). Work-family imbalance has been reported to be a risk factor for depression (Frone et al. 1996), reduced well-being (Grant-Vallone and Donaldson 2001), exhaustion (Demerouti et al. 2004), and alcohol abuse (Wang et al. 2010). The work-life interface has also been reported to be related to sleep. Those who had difficulties combining work and private life had increased odds for sleep disorders (men adjusted OR 1.54, 95 % CI 1.12–2.10 and women adjusted OR 1.81, 95 % CI 1.31–2.49) (Hammig and Bauer 2009). Another study in medical residents showed that work-family conflict was associated with sleep deprivation (Geurts et al. 1999). Our study found that work-life imbalance is related to increased sleep problems in Korean workers as well.

Job satisfaction has been consistently associated with sleep problems in earlier studies (Doi et al. 2003; Kuppermann et al. 1995; Nakata et al. 2004a, 2007, 2008; Scott and Judge 2006). The results of our study are in line with these findings. For example, Scott and Judge (2006) reported that insomnia is positively related to job dissatisfaction and this relationship is mediated by hostility, joviality, and attentiveness in US administrative employees (Scott and Judge 2006). Doi et al. (2003) found that job dissatisfaction is the second major factor for poor sleep quality, which resulted in a twofold increase in the prevalence of disturbed sleep among white-collar employees in Japan (Doi et al. 2003). Another study in Japan revealed that low job satisfaction created a significantly increased risk for insomnia including difficulty maintaining sleep (DMS) after adjusting for multiple confounding factors (Nakata et al. 2004a). Our study, together with those from other countries, indicates that job dissatisfaction is a risk factor associated with sleep problems.

High cognitive and emotional demands, as well as high work intensity, increased the risk of sleep problems. A significant association between cognitive demands and difficulty initiating sleep (DIS) was found in male white-collar daytime workers in Japan (Nakata et al. 2004a). Urponen et al. (1988) also reported that mental workload was one of the most important factors that interfered with falling asleep (Urponen et al. 1988). In terms of work intensity, there is consensus that high job demands are related to insomnia (Cahill and Landsbergis 1996; Kalimo et al. 2000; Pelfrene et al. 2002). Excessive mental/cognitive demands and working too hard may disturb the ability to fall asleep, which in turn may impair the quality of sleep.

In our study, social support at work was not associated with sleep problems after adjusting for confounding factors. Although the majority of published studies (Cahill and Landsbergis 1996; Eriksen et al. 2008; Jansson and Linton 2006; Kageyama et al. 1998; Kim et al. 2011; Nakata et al. 2001, 2007; Nordin et al. 2005; Pelfrene et al. 2002; Runeson et al. 2011) indicate that poor social support at work is related to sleep problems, some studies suggest that the statistical significance of this relationship is attenuated after controlling for confounders (Nakata et al. 2004a, 2006, 2008). This finding may be relevant to the fact that social support often exerts a buffering effect on health outcomes and that the significant relationship disappears if controlled for related variables. However, it is important to note that social support from one’s workplace is often more protective than social support from family or friends, suggesting the importance of workplace social support (Nakata et al. 2001, 2004a).

A significant association between job insecurity and sleep problems was found in this study. After the 1998 financial crisis in East Asia, Korea was no exception with regard to increased job insecurity. At the time of the crisis, a large number of workers lost their jobs and since then businesses have not been active in recruiting permanent employees (preferring temporary employees), and employers are facing organizational restructuring over time. Workers who feel their jobs are insecure may succumb to sleep disorders resulting in long-term mental stress. A study of civil servants in Britain reported that male workers who experienced organizational change tended to have increased sleep problems (Ferrie et al. 1998). Another Swedish study discovered that workers who expected that they would lose their jobs experienced sleep disturbances (Mattiasson et al. 1990). The results of this study support the notion that job insecurity is connected to sleep problems.

The overall prevalence of WRSP in this study was 5.1 %, which was comparable to that of 8.7 % in the fourth EWCS (Table 3). The sleep problems question used in both the KWCS and the EWCS was targeted specifically to work-related sleep problems. A study in Sweden (Swedish Work Environment Survey; SWES) used a similar method to define sleep disturbances as both the KWCS and EWCS and showed a strong predictability of medically certified sickness absence (Westerlund et al. 2008). In comparison with earlier studies that defined sleep problems in general, the definition used in the KWCS, EWCS, and SWES might have a stronger predictive validity than merely asking about general sleep problems because general sleep problems may also capture problems related to or caused by non-work-related issues. However, it is also true that the significant associations found in this study are subject to the ‘triviality trap’; that is the measurement of the independent (WRSP) and dependent (organization factors) variables is conceptually overlapping and the observed associations may be spurious (Kristensen 1996). Thus, future studies should be undertaken to validate our finding by using objective sleep measures in a prospective study design.

The analyses of underlying factors associated with WRSP revealed that men had a 1.5 times higher odds of WRSP than women (Table 4). In studies investigating sex differences in sleep problems, the majority of studies discovered that sleep problems are more frequent in women than in men (Chen et al. 2005; Kim et al. 2011; Paparrigopoulos et al. 2010). However, in this study, as the definition of sleep problems was ‘work-related,’ it may be that working men in Korea have more sleep problems due to work than working women do. In the EWCS, the prevalence of sleep problems in men was 8.9 %, while it was 8.5 % in women. Thus, it is likely that the higher prevalence of sleep problems in men than in women may depend on how ‘sleep problems’ are defined. As suggested in Table 4, the higher prevalence of WRSP in workers with illness and working the shift/night schedule is in line with previous findings, indicating that the association was in the expected direction.

### Strengths and limitations of the study

The specific strengths of this study are that: (a) the sample was both nationally representative of the Korean working population and was large in size, (b) the study measured a number of work organization factors, (c) the analyses controlled for a broad array of potential confounders related to work organization and sleep problems, and d) the survey measures were collected via face-to-face interviews resulting in very little missing data. A major criticism of the methodology of the present study is that we evaluated WRSP with a single question, which prevented us from judging the severity of sleep problems and did not allow us to compare our results with other studies that used more general questions. Moreover, the definition of WRSP may include not only those with general sleep problems, that is, insomnia, poor sleep quality, and sleep loss, but also those with more specific sleep disorders, that is, sleep apnea, excessive daytime sleepiness, severe bruxism, etc. We also acknowledge other potential limitations. First, the study is cross-sectional in nature; thus, no causal interpretations can be made. However, it may be speculated that sleep problems affect the rating of work conditions; workers with sleep problems may have issues with irritability with colleagues and supervisors, an inability to concentrate at work, difficulty accomplishing assigned tasks in a timely manner, and uncertainty that they will be able to continue their employment, leading to expressions of higher work stress (Nakata et al. 2007). Meanwhile, poor working conditions may influence sleep problems. A two-year prospective study of the effort-reward imbalance model, the job demand-control model, and insomnia revealed that those who were not insomniac at the baseline became insomniac when exposed to high overcommitment to work (OR 1.75, *p* < 0.05) and high job strain (OR 1.72, *p* < 0.05) (Ota et al. 2009). Second, most of the work organization measures consisted of single item that may raise questions as to the validity and reliability of the results. However, items such as ‘job satisfaction’ are known to hold as high a reliability as multi-item scales (Wanous et al. 1997). Third, even though we have statistically controlled for existing disorders, it is possible that those who are suffering from sleep problems may be affected by comorbid disorders.

## Conclusions

This study found a significant relationship between a broad range of work organization characteristics and sleep problems, which has been understudied in representative samples of workers. Although a prospective study with objective sleep measures is warranted to prevent the ‘triviality trap,’ the present finding that work organization factors are related to sleep problems may be useful in developing strategies to prevent sleep problems in the Korean working population.

## References

[CR1] Akerstedt T, Fredlund P, Gillberg M, Jansson B (2002). Work load and work hours in relation to disturbed sleep and fatigue in a large representative sample. J Psychosom Res.

[CR2] Akerstedt T, Kecklund G, Selen J (2010). Disturbed sleep and fatigue as predictors of return from long-term sickness absence. Ind Health.

[CR3] Ballard TJ, Romito P, Lauria L (2006). Self perceived health and mental health among women flight attendants. Occup Environ Med.

[CR4] Bowling A (2005). Mode of questionnaire administration can have serious effects on data quality. J Public Health (Oxf).

[CR5] Cahill J, Landsbergis PA (1996). Job strain among post office mailhandlers. Int J Health Serv.

[CR6] Chen YY, Kawachi I, Subramanian SV, Acevedo-Garcia D, Lee YJ (2005). Can social factors explain sex differences in insomnia? Findings from a national survey in Taiwan. J Epidemiol Community Health.

[CR7] Cho YW, Shin WC, Yun CH, Hong SB, Kim J, Earley CJ (2009). Epidemiology of insomnia in korean adults: prevalence and associated factors. J Clin Neurol.

[CR8] Dahlgren A, Kecklund G, Akerstedt T (2006). Overtime work and its effects on sleep, sleepiness, cortisol and blood pressure in an experimental field study. Scand J Work Environ Health.

[CR9] Demerouti E, Geurts SA, Bakker AB, Euwema M (2004). The impact of shiftwork on work–home conflict, job attitudes and health. Ergonomics.

[CR10] Doi Y, Minowa M, Tango T (2003). Impact and correlates of poor sleep quality in Japanese white-collar employees. Sleep.

[CR11] Elovainio M, Ferrie JE, Gimeno D (2009). Organizational justice and sleeping problems: the Whitehall II study. Psychosom Med.

[CR12] Eriksen W, Bjorvatn B, Bruusgaard D, Knardahl S (2008). Work factors as predictors of poor sleep in nurses’ aides. Int Arch Occup Environ Health.

[CR13] Estryn-Behar M, Kaminski M, Peigne E (1990). Stress at work and mental health status among female hospital workers. Br J Ind Med.

[CR14] Fahlen G, Knutsson A, Peter R (2006). Effort-reward imbalance, sleep disturbances and fatigue. Int Arch Occup Environ Health.

[CR15] Ferrie JE, Shipley MJ, Marmot MG, Stansfeld S, Davey Smith G (1998). The health effects of major organisational change and job insecurity. Soc Sci Med.

[CR16] Frone MR, Russell M, Barnes GM (1996). Work-family conflict, gender, and health-related outcomes: a study of employed parents in two community samples. J Occup Health Psychol.

[CR17] Geurts S, Rutte C, Peeters M (1999). Antecedents and consequences of work-home interference among medical residents. Soc Sci Med.

[CR18] Goldstein TR, Bridge JA, Brent DA (2008). Sleep disturbance preceding completed suicide in adolescents. J Consult Clin Psychol.

[CR19] Gradus JL, Street AE, Kelly K, Stafford J (2008). Sexual harassment experiences and harmful alcohol use in a military sample: Differences in gender and the mediating role of depression. J Stud Alcohol Drugs.

[CR20] Grant-Vallone EJ, Donaldson SI (2001). Consequences of work-family conflict on employee well-being over time. Work Stress.

[CR21] Hall JK, Spector PE (1991). Relationships of work stress measures for employees with the same job. Work Stress.

[CR22] Hammig O, Bauer G (2009). Work-life imbalance and mental health among male and female employees in Switzerland. Int J Public Health.

[CR23] Jansson M, Linton SJ (2006). Psychosocial work stressors in the development and maintenance of insomnia: a prospective study. J Occup Health Psychol.

[CR24] Kageyama T, Nishikido N, Kobayashi T, Kurokawa Y, Kaneko T, Kabuto M (1998). Self-reported sleep quality, job stress, and daytime autonomic activities assessed in terms of short-term heart rate variability among male white-collar workers. Ind Health.

[CR25] Kalimo R, Tenkanen L, Harma M, Poppius E, Heinsalmi P (2000). Job stress and sleep disorders: findings from the Helsinki Heart Study. Stress Med.

[CR26] Kessler RC, Mickelson KD, Williams DR (1999). The prevalence, distribution, and mental health correlates of perceived discrimination in the United States. J Health Soc Behav.

[CR27] Kim HC, Kim BK, Min KB, Min JY, Hwang SH, Park SG (2011). Association between job stress and insomnia in Korean workers. J Occup Health.

[CR28] Kling RN, McLeod CB, Koehoorn M (2010). Sleep problems and workplace injuries in Canada. Sleep.

[CR29] Knudsen HK, Ducharme LJ, Roman PM (2007). Job stress and poor sleep quality: data from an American sample of full-time workers. Soc Sci Med.

[CR30] Kristensen TS (1996). Job stress and cardiovascular disease: a theoretic critical review. J Occup Health Psychol.

[CR31] Kubota K, Shimazu A, Kawakami N, Takahashi M, Nakata A, Schaufeli WB (2010). Association between workaholism and sleep problems among hospital nurses. Ind Health.

[CR32] Kudielka BM, Von Kanel R, Gander ML, Fischer JE (2004). Effort-reward imbalance, overcommitment and sleep in a working population. Work Stress.

[CR33] Kuppermann M, Lubeck DP, Mazonson PD (1995). Sleep problems and their correlates in a working population. J Gen Intern Med.

[CR34] Lallukka T, Rahkonen O, Lahelma E (2011). Workplace bullying and subsequent sleep problems–the Helsinki Health Study. Scand J Work Environ Health.

[CR35] Leger D, Bayon V (2010). Societal costs of insomnia. Sleep Med Rev.

[CR36] Lombardi DA, Folkard S, Willetts JL, Smith GS (2010). Daily sleep, weekly working hours, and risk of work-related injury: US national health interview survey (2004–2008). Chronobiol Int.

[CR37] Mallon L, Broman JE, Hetta J (2002). Sleep complaints predict coronary artery disease mortality in males: a 12-year follow-up study of a middle-aged Swedish population. J Intern Med.

[CR38] Mattiasson I, Lindgarde F, Nilsson JA, Theorell T (1990). Threat of unemployment and cardiovascular risk factors; longitudinal study of quality of sleep and serum-cholesterol concentrations in men threatened with redundancy. B Med J.

[CR39] Metlaine A, Leger D, Choudat D (2005). Socioeconomic impact of insomnia in working populations. Ind Health.

[CR40] Motohashi Y, Takano T (1995). Sleep habits and psychosomatic health complaints of bank workers in a megacity in Japan. J Biosoc Sci.

[CR41] Murata C, Yatsuya H, Tamakoshi K, Otsuka R, Wada K, Toyoshima H (2007). Psychological factors and insomnia among male civil servants in Japan. Sleep Med.

[CR42] Nakata A (2011). Effects of long work hours and poor sleep characteristics on workplace injury among full-time male employees of small- and medium-scale businesses. J Sleep Res.

[CR43] Nakata A (2011). Work hours, sleep sufficiency, and prevalence of depression among full-time employees: a community-based cross-sectional study. J Clin Psychiatry.

[CR44] Nakata A, Haratani T, Takahashi M (2001). Job stress, social support at work, and insomnia in Japanese shift workers. J Hum Ergol (Tokyo).

[CR45] Nakata A, Haratani T, Takahashi M (2004). Job stress, social support, and prevalence of insomnia in a population of Japanese daytime workers. Soc Sci Med.

[CR46] Nakata A, Haratani T, Takahashi M (2004). Association of sickness absence with poor sleep and depressive symptoms in shift workers. Chronobiol Int.

[CR47] Nakata A, Ikeda T, Takahashi M (2006). Impact of psychosocial job stress on non-fatal occupational injuries in small and medium-sized manufacturing enterprises. Am J Ind Med.

[CR48] Nakata A, Takahashi M, Ikeda T, Haratani T, Hojou M, Araki S (2007). Perceived job stress and sleep-related breathing disturbance in Japanese male workers. Soc Sci Med.

[CR49] Nakata A, Takahashi M, Ikeda T, Hojou M, Araki S (2008). Perceived psychosocial job stress and sleep bruxism among male and female workers. Community Dent Oral Epidemiol.

[CR50] Nena E, Steiropoulos P, Constantinidis TC, Perantoni E, Tsara V (2010). Work productivity in obstructive sleep apnea patients. J Occup Environ Med.

[CR51] Niedhammer I, Lert F, Marne MJ (1994). Effects of shift work on sleep among French nurses. A longitudinal study. J Occup Med.

[CR52] Niedhammer I, David S, Degioanni S (2009). Workplace bullying and sleep disturbances: findings from a large scale cross-sectional survey in the French working population. Sleep.

[CR53] Nomura K, Yamaoka K, Nakao M, Yano E (2010). Social determinants of self-reported sleep problems in South Korea and Taiwan. J Psychosom Res.

[CR54] Nordin M, Knutsson A, Sundbom E, Stegmayr B (2005). Psychosocial factors, gender, and sleep. J Occup Health Psychol.

[CR55] Ohayon MM (2002). Epidemiology of insomnia: what we know and what we still need to learn. Sleep Med Rev.

[CR56] Ohayon MM, Hong SC (2002). Prevalence of insomnia and associated factors in South Korea. J Psychosom Res.

[CR57] Ota A, Masue T, Yasuda N, Tsutsumi A, Mino Y, Ohara H (2005). Association between psychosocial job characteristics and insomnia: an investigation using two relevant job stress models–the demand-control-support (DCS) model and the effort-reward imbalance (ERI) model. Sleep Med.

[CR58] Ota A, Masue T, Yasuda N (2009). Psychosocial job characteristics and insomnia: a prospective cohort study using the Demand-Control-Support (DCS) and Effort-Reward Imbalance (ERI) job stress models. Sleep Med.

[CR59] Paparrigopoulos T, Tzavara C, Theleritis C, Psarros C, Soldatos C, Tountas Y (2010). Insomnia and its correlates in a representative sample of the Greek population. BMC Public Health.

[CR60] Parent-Thirion A, Fernández Macías E, Hurley J, Vermeylen G (2006) Fourth European working conditions survey

[CR61] Park BJ, Lee N (2006) First Korean Working Conditions Survey. Occupational Safety and Health Research Institute, Korean Occupational Safety and Health Agency (KOSHA), Incheon

[CR62] Park J, Lee N (2009). First Korean Working Conditions Survey: a comparison between South Korea and EU countries. Ind Health.

[CR63] Park J, Yi Y, Kim Y (2010). Weekly work hours and stress complaints of workers in Korea. Am J Ind Med.

[CR64] Pavalko EK, Mossakowski KN, Hamilton VJ (2003). Does perceived discrimination affect health? Longitudinal relationships between work discrimination and women’s physical and emotional health. J Health Soc Behav.

[CR65] Pelfrene E, Vlerick P, Kittel F, Mak RP, Kornitzer M, De Backer G (2002). Psychosocial work environment and psychological well-being: assessment of the buffering effects in the job demand-control (-support) model in BELSTRESS. Stress Health.

[CR66] Philip P, Taillard J, Niedhammer I, Guilleminault C, Bioulac B (2001). Is there a link between subjective daytime somnolence and sickness absenteeism? A study in a working population. J Sleep Res.

[CR67] Rogers KA, Kelloway EK (1997). Violence at work: personal and organizational outcomes. J Occup Health Psychol.

[CR68] Rosekind MR, Gregory KB, Mallis MM, Brandt SL, Seal B, Lerner D (2010). The cost of poor sleep: workplace productivity loss and associated costs. J Occup Environ Med.

[CR69] Runeson R, Lindgren T, Wahlstedt K (2011). Sleep problems and psychosocial work environment among Swedish commercial pilots. Am J Ind Med.

[CR70] Salminen S, Oksanen T, Vahtera J (2010). Sleep disturbances as a predictor of occupational injuries among public sector workers. J Sleep Res.

[CR71] Scott BA, Judge TA (2006). Insomnia, emotions, and job satisfaction: a multilevel study. J Manage.

[CR72] Sinokki M, Ahola K, Hinkka K (2010). The association of social support at work and in private life with sleeping problems in the Finnish health 2000 study. J Occup Environ Med.

[CR73] Soldatos CR, Allaert FA, Ohta T, Dikeos DG (2005). How do individuals sleep around the world? Results from a single-day survey in ten countries. Sleep Med.

[CR74] Statistics Korea (2007) Korean Standard Classification of Occupations (KSCO)

[CR75] Street AE, Stafford J, Mahan CM, Hendricks A (2008). Sexual harassment and assault experienced by reservists during military service: Prevalence and health correlates. J Rehabil Res Dev.

[CR76] Takaki J, Taniguchi T, Fukuoka E (2010). Workplace bullying could play important roles in the relationships between job strain and symptoms of depression and sleep disturbance. J Occup Health.

[CR77] Urponen H, Vuori I, Hasan J, Partinen M (1988). Self-evaluations of factors promoting and disturbing sleep: an epidemiological survey in Finland. Soc Sci Med.

[CR78] Vahtera J, Pentti J, Helenius H, Kivimaki M (2006). Sleep disturbances as a predictor of long-term increase in sickness absence among employees after family death or illness. Sleep.

[CR79] Wang M, Liu S, Zhan Y, Shi J (2010). Daily work-family conflict and alcohol use: testing the cross-level moderation effects of peer drinking norms and social support. J Appl Psychol.

[CR80] Wanous JP, Reichers AE, Hudy MJ (1997). Overall job satisfaction: how good are single-item measures?. J Appl Psychol.

[CR81] Weissman MM, Greenwald S, Nino-Murcia G, Dement WC (1997). The morbidity of insomnia uncomplicated by psychiatric disorders. Gen Hosp Psychiatry.

[CR82] Westerlund H, Alexanderson K, Akerstedt T, Magnusson Hanson L, Theorell T, Kivimaki M (2008). Work-related sleep disturbances and sickness absence in the Swedish working population, 1993–1999. Sleep.

[CR83] Yang H, Schnall PL, Jauregui M, Su TC, Baker D (2006). Work hours and self-reported hypertension among working people in California. Hypertension.

